# Characterization of Magnetorheological Impact Foams in Compression

**DOI:** 10.3390/mi15060782

**Published:** 2024-06-14

**Authors:** Young Choi, Norman M. Wereley

**Affiliations:** Department of Aerospace Engineering, University of Maryland, College Park, MD 20742, USA; nicechoi@umd.edu

**Keywords:** adaptive cushioning material, compressive property, impact, isotropic, energy absorption and dissipation, magnetorheological elastomeric foam (MREF)

## Abstract

This study focuses on the development and compressive characteristics of magnetorheological elastomeric foam (MREF) as an adaptive cushioning material designed to protect payloads from a broader spectrum of impact loads. The MREF exhibits softness and flexibility under light compressive loads and low strains, yet it becomes rigid in response to higher impact loads and elevated strains. The synthesis of MREF involved suspending micron-sized carbonyl Fe particles in an uncured silicone elastomeric foam. A catalyzed addition crosslinking reaction, facilitated by platinum compounds, was employed to create the rapidly setting silicone foam at room temperature, simplifying the synthesis process. Isotropic MREF samples with varying Fe particle volume fractions (0%, 2.5%, 5%, 7.5%, and 10%) were prepared to assess the effect of particle concentrations. Quasi-static and dynamic compressive stress tests on the MREF samples placed between two multipole flexible strip magnets were conducted using an Instron servo-hydraulic test machine. The tests provided measurements of magnetic field-sensitive compressive properties, including compression stress, energy absorption capability, complex modulus, and equivalent viscous damping. Furthermore, the experimental investigation also explored the influence of magnet placement directions (0° and 90°) on the compressive properties of the MREFs.

## 1. Introduction

Flexible elastomeric foams can be found in extensive applications as packaging and cushioning materials across various engineering fields and sports domains. Their popularity stems from their lightweight nature, straightforward configuration, and flexibility in geometric design. These foams offer effective vibration damping, sound and thermal insulation, and serve as protective barriers for delicate instruments, safeguarding them from impacts and mitigating the risk of severe injury to individuals in critical areas during collisions and falls.

The crashworthiness protection effectiveness of flexible elastomeric foams is heavily reliant on their ability to absorb and dissipate energy. However, due to their relatively lower generated force levels and limited energy absorption and dissipation capacities, these conventional foams are primarily effective in providing protection against low-velocity impacts. Enhancing crashworthiness performance for higher-velocity impacts requires designing flexible elastomeric foams in thicker and denser forms, making them less flexible, stiffer, heavier, and ultimately compromising their protection capabilities against low-velocity impacts. This drawback can be overcome by employing adaptive cushioning materials that are soft and flexible under light compressive loads and low strain but stiffen when subjected to higher impact loads and increased strains [1,2]. Such an adaptive cushioning material can be implemented by using magnetorheological elastomeric foams (MREFs).

MREFs are a class of magnetorheological (MR) materials that are also called smart materials having the capability of changing their mechanical properties to cope with various working environments. One of the representative MR materials is MR fluids comprised of randomly dispersed magnetic particles in the carrier fluid. In the absence of a magnetic field, the magnetic particles of MR fluids are freely movable but when a magnetic field is applied they form chain-like particle columns in parallel to the magnetic field direction. As a result, their rheological properties such as the fluid viscosity and yield stress can be continuously, rapidly, and reversibly changed by an applied magnetic field. Because of these features, MR fluids have been actively developed for various hydraulic actuator systems [3,4] and shock and vibration mitigation systems [5,6,7]. However, since the large density difference between the magnetic particles of MR fluids and the carrier fluids [8,9], MR fluids inevitably encounter a certain level of sedimentation issues after long-term downtime. Also, a certain level of sealing is demanded to prevent fluid leakage [10]. MREFs consist of magnetic particles permanently dispersed in a matrix of elastomeric foams. Thus, MREFs are inherently free of sedimentation and fluid leakage and show larger controllable stiffness and damping capabilities because of very soft and flexible in the absence of a magnetic field. These advantages have prompted researchers to investigate the application of adaptively controllable vibration and sound absorption devices [11,12,13,14,15] and a haptic tactile device [16].

This study focused on developing MREFs by placing them between flexible strip magnets to activate them passively. These MREFs were aimed to serve as adaptive cushioning materials, and their compressive characteristics were examined through experimental methods. The MREFs were created by dispersing micron-sized carbonyl Fe particles within uncured silicone elastomeric foam. Various samples with different volume fractions of Fe particles (ranging from 0% to 10%) were fabricated to assess the impact of particle concentrations. The compressive properties of these MREF samples were evaluated under both quasi-static and dynamic conditions using a servo-hydraulic test machine. Performance parameters such as compression stress, energy absorption, complex modulus, and equivalent viscous damping were measured to evaluate the magnetic field-sensitive mechanical properties of the materials. Furthermore, the study investigated how the orientation of the magnet placement (either at 0° or 90°) affected the compressive properties of the MREFs through experimental analysis.

## 2. Experimental

### 2.1. Materials

**Figure 1:**
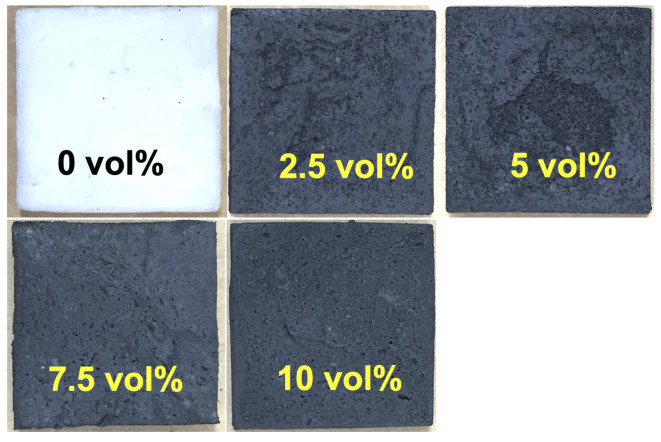
Square-shaped isotropic magnetorheological elastomeric foam (MREF) samples fabricated with different carbonyl Fe particle concentrations (nominal size: 50.8 mm × 50.8 mm × 12.7 mm).

Figure 1 presents isotropic MREF samples created with varying concentrations of carbonyl Fe particles. Here, vol% indicates volume fraction. In this study, a platinum-catalyzed addition crosslinking reaction was employed to generate a rapid-setting silicone foam (Smooth-On Inc., Macungie, PA, USA, Soma Foama 15) at room temperature, providing a pot life of less than 1 min. The liquid silicone foam consists of parts A and B to prevent catalyzing reactions. Parts A and B were dispensed into a 3D-printed mold in which carbonyl Fe particles were already added. After that, the dispensed liquid silicone foam was thoroughly mixed for 30 s and cured for 1 h. This method significantly simplified the synthesis of these MREF materials. It is noteworthy that silicone foams offer advantages such as a broader range of attainable mechanical properties and superior resistance to heat, chemicals, ozone, ultraviolet (UV) radiation, and weathering [17]. Furthermore, silicone foams exhibit a lower compression set, indicating the extent of permanent deformation after the removal of compressive stress, and demonstrate superior longevity. Carbonyl Fe powders (BASF Corp., Ludwigshafen, Germany), ranging from 1 to 10 microns in size, with particle concentrations of 0–10 vol%, were dispersed as Fe particles. The fabricated MREF samples in this study were isotropic magnetic materials, where the Fe particles were dispersed randomly in the silicone foam without the influence of a magnetic field, ensuring no specific orientation [18,19]. The MREF samples were square in shape, with nominal dimensions of 50.8 mm × 50.8 mm × 12.7 mm (2 in × 2 in × 0.5 in). To induce a magnetic field for activating the Fe particles in the MREF samples, two flexible commercial strip magnets, composed of neodymium-Fe-boron magnetic powders bonded with synthetic rubber and featuring a multipole magnetization pattern on one face were positioned on the top and bottom of the MREF samples. It is important to highlight that the flexible multiple-strip magnet employed in this study featured an arrangement of north and south magnetic poles positioned side-by-side on one face. As a result, the magnetic flux of this magnet does not extend far from its surface but can be more potent compared to general flexible dipole magnets. The specified dimensions of the flexible strip magnet were 50.8 mm × 50.8 mm × 1.6 mm (2 in × 2 in × 0.0625 in).

**Figure 2:**
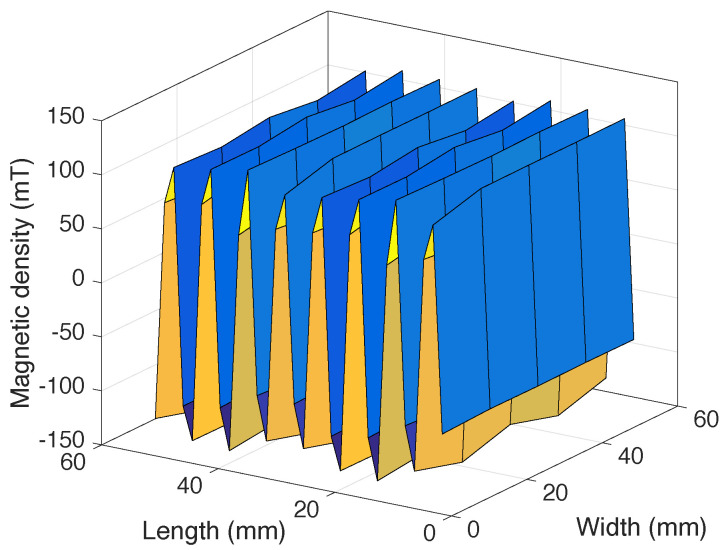
Magnetic density of the flexible multipole strip magnet with respect to the length and width ($B_{RMS}$ = 104 mT and 8 poles per inch).

Figure 2 presents the measured magnetic density of the flexible multipole strip magnet concerning its length and width. A gauss/teslameter (F.W. Bell, 5080, Portland, OR, USA) was employed for measuring the magnetic density at the surface of the strip magnet, where positive magnetic density indicates the north magnetic pole and negative denotes the south magnetic pole. As presented in the figure, the flexible strip magnet exhibited a multipole configuration along its length with an 8 poles per inch (PPI) array, and the measured root-mean-square (RMS) magnetic density level was $B_{\mathrm{RMS}}$ = 104 mT.

### 2.2. Compressive Test Setup

**Figure 3:**
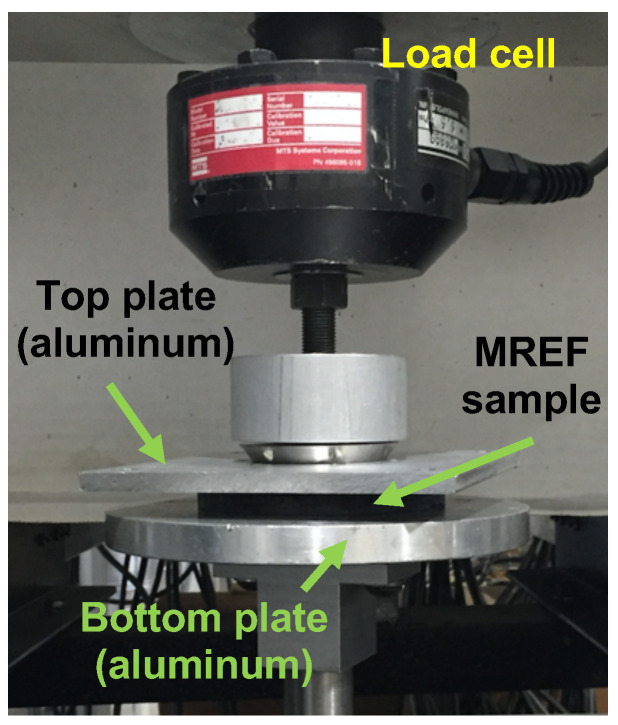
Experimental setup of the compressive test of the MREF samples by using an Instron servo-hydraulic test machine.

**Figure 4:**
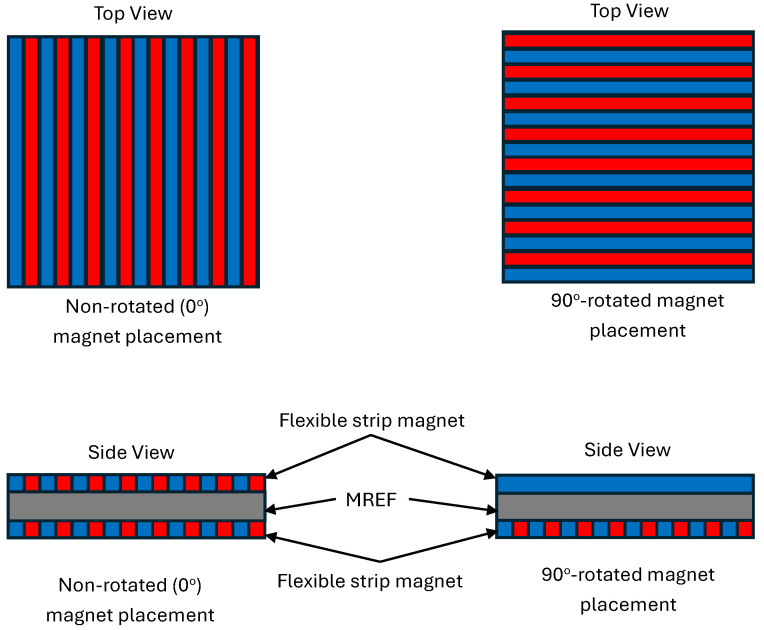
Schematic diagram for the magnetic placement directions for the MREF samples. Here, the blue and red colors mean the magnetic poles.

Figure 3 presents the experimental setup for the compressive tests conducted on the MREF samples using a Dynomite servo-hydraulic machine (Instron, Norwood, MA, USA). The top plate was affixed to the load cell, while the bottom plate was connected to the servo-hydraulic actuator. Both plates in this study were constructed from nonmagnetic aluminum material to ensure that the magnetic field from the flexible strip magnets permeated the MREF sample more effectively and did not escape into the plates. The MREF sample, positioned between two flexible strip magnets, was situated between the top and bottom plates. External compression force was applied by the actuator through the bottom plate, and the load cell measured the resistive force of the MREF sample during compressive strain. The study encompassed quasi-static and dynamic compressive tests on MREF samples with different particle concentrations to assess the impact of particle concentrations. Moreover, as presented in Figure 2, given the multipole magnetization pattern of the flexible strip magnets, the study explored the influence of magnet placement directions. Figure 4 presents the schematic diagram for the magnetic placement directions for the MREF samples. One configuration involved non-rotated (0°) magnet placement, where the top and bottom magnets exhibited the same multipole direction. The other configuration entailed a 90°-rotated magnet placement, in which the multipole direction of the top and bottom magnets displayed a 90° difference.

## 3. Results and Discussion

### 3.1. Quasi-Static Compressive Tests

**Figure 5:**
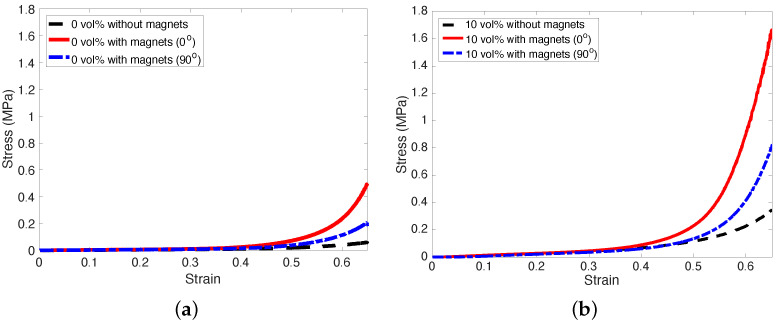
Compressive stress versus strain curves for isotropic MREF samples with Fe particle concentrations of 0 and 10 vol%. (**a**) 0 vol% (No Fe particle); (**b**) 10 vol%.

In this study, the quasi-static compressive tests of the MREFs were conducted up to 65% strain with a strain rate of 0.4 mm/s. The compressive stress versus strain curves for the isotropic MREF samples with Fe particle concentrations of 0 and 10 vol% were presented in Figure 5. In this case, “with magnets (0°)” implies the no-rotated magnet placement case, and “with magnets (90°)” means the 90°-rotated magnet placement case. Also, the MREF with 0 vol% implies a pure silicone foam. Thus, as observed in Figure 5a, the MREF with 0 vol% and no magnets showed a very low stress level even though it compressed up to 65% strain. The silicone foam utilized as the base material for the MREFs in this study exhibited significant softness and flexibility. However, with the introduction of two flexible strip magnets, the compressive stress of the silicone foam increased notably beyond a strain of 40%. This elevation in compressive stress arises from the repulsive force generated by the magnets. As the repulsive magnetic force follows an inverse square relationship with distance, the presence of magnets enabled the silicone foam to exhibit higher compressive stress levels at greater strains compared to cases without magnets. In contrast, the case where magnets were placed without rotation (0°) displayed a higher level of compressive stress than the case where magnets were rotated at 90°. This discrepancy is attributable to the alignment of magnetic poles. In the non-rotated magnet placement, the identical magnetic poles of the top and bottom magnets are positioned 180° apart from each other. Conversely, in the 90°-rotated placement, only half of the corresponding magnetic poles directly oppose each other, resulting in a reduced repulsive magnetic force. Consequently, the non-rotated magnet placement case generated a higher repulsive force compared to the 90°-rotated placement.

On the other hand, the compressive test results for MREFs with a 10 vol% composition were depicted in Figure 5b. As illustrated, up to 35% strain, the compressive stresses of the MREFs with 10 vol% remained low, indicating a soft behavior. However, beyond 35% strain, the compressive stresses experienced a significant increase. Both the MREFs with magnets (0° and 90°) exhibited notably higher compressive stresses compared to the MREFs without magnets. This escalation in compressive stress for MREFs with 10 vol% can be attributed to two factors: the repulsive magnetic force and the alignment of Fe magnetic particles (i.e., MR effect) induced by the magnetic field within the matrix. As expected from the trend in Figure 5a, the MREF with magnets (0°) showed a higher compressive stress level than the MREF with magnets (90°).

**Figure 6:**
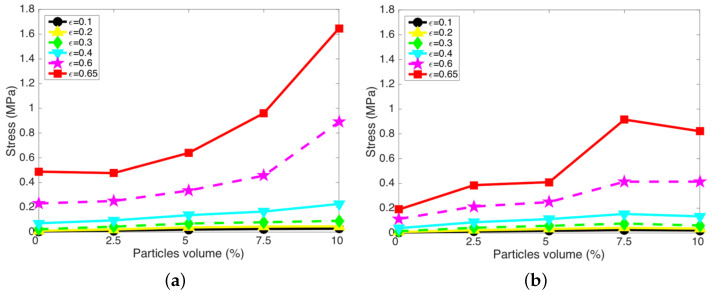
Compressive stress versus particle concentration for the MREFs for a range of discrete strain (ϵ) levels. (**a**) with magnets (0°); (**b**) with magnets (90°).

Figure 6 presents compressive stress versus particle concentration for the MREFs for a range of discrete strain (ϵ) levels. As seen in this figure, compressive stresses exhibit a highly nonlinear dependence on both strain and particle concentration, with an increase observed as particle concentration and strain rise. Notably, across all tested particle concentrations, the case with no-rotated magnet placement consistently displayed higher compressive stress levels compared to the 90°-rotated magnet placement case. The greatest disparity in compressive stress levels between the two magnet placement configurations occurred at a particle concentration of 10 vol%.

**Figure 7:**
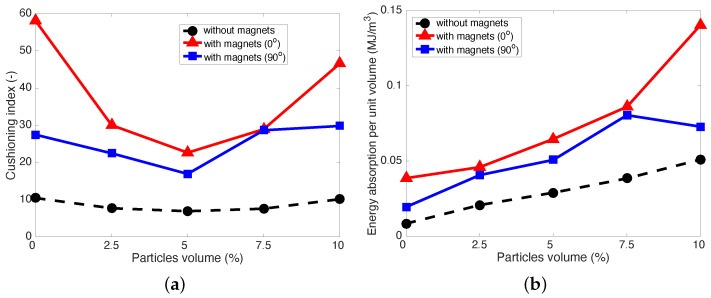
Cushioning index and energy absorption density of the MREFs versus particle concentration. (**a**) cushioning index; (**b**) energy absorption density.

Figure 7 presents the cushioning index and energy absorption density of the MREFs versus the particle concentration. In this study, the cushioning index, CI, was defined by the ratio of the compressive stress level at 65% strain to the compressive stress level at 25% strain like

(1) $$\;CI=\frac{\sigma_{\epsilon =65}}{\sigma_{\epsilon =25}}$$

Here, $\sigma_{\epsilon =\mathrm{xx}}$ is the compressive stress at xx% strain, ϵ=xx/100. A higher cushioning index, CI implies that foams are soft and flexible at lower strains but become firmer and stiffer at higher strains to prevent from bottoming out. On the other hand, the energy absorption density, $W_{s}$, was calculated by

(2) $$\;W_{s}=\int_{0}^{\epsilon }\sigma \;d\epsilon \;$$

Here, σ is the stress and ϵ is the strain. This energy absorption density, $W_{s}$, signifies the amount of energy absorbed by the material per unit volume during deformation. As presented in Figure 7a, the cushioning indices of the MREFs initially exhibited a decline with increasing particle concentration, but beyond 5 vol%, there was a subsequent rise in their cushioning indices. This indicates that up to 5 vol%, the augmentation of particle concentration resulted in an elevation of compressive stress at low strain rather than at high strain. This phenomenon is likely attributed to the particle-matrix interaction in enhancing the compressive stress of MREFs at lower Fe particle concentrations. However, at higher Fe particle concentrations exceeding 5 vol%, the reinforcement of MREFs may stem not only from particle-matrix interaction but also from the particle-particle jamming effect [20,21,22]. Consequently, the increase in compressive stress of MREFs due to Fe particle concentration became more pronounced at high strain compared to low strain. The variation in cushion index between no magnets and with magnets was most prominent in the case with non-rotated magnetic placement, which also exhibited the highest cushioning index. On the other hand, the energy absorption densities of the MREFs exhibited an almost continuous increase with particle concentration, with the non-rotated magnetic placement case again demonstrating the highest energy absorption density.

### 3.2. Dynamic Compression Tests

Dynamic compression tests of the MREFs were conducted by using sinusoidal strain inputs that had 10% strain amplitude and their frequencies were swept from 1 Hz to 20 Hz with a 1 Hz increment. In this case, the MREFs were initially compressed by 50% strain. The complex modulus, *Z*, and the equivalent viscous damping, $C_{eq}$ were used to evaluate the mechanical properties of the MREFs under dynamic compressive tests.

The complex modulus was used to assess the dynamic characteristics of these nonlinear materials. It is a technique utilized in the frequency domain, primarily aimed at linearizing such materials for analysis purposes. In the complex modulus analysis, the nonlinear hysteresis stress–strain loop is replaced with an equivalent elliptical viscoelastic stress–strain loop. The complex modulus [23,24,25], *Z*, was given by

(3) $$\;Z=Z^{\prime }+jZ^{\prime \prime }$$

Here, *j* is the imaginary number. $Z^{\prime }$ is the storage modulus, which implies the ability of the material to store potential energy and release it over a deformation cycle and $Z^{\prime \prime }$ is the loss modulus, which is associated with the ability of the material to dissipate the energy over a deformation cycle. Using the storage modulus and loss modulus, the loss factor, η, is given by

(4) $$\eta =\frac{Z^{\prime \prime }}{Z^{\prime }}$$

As seen Equation (4), the loss factor is the ratio of the loss modulus to storage modulus in a material, which provides a measure of damping in the material. On the other hand, the equivalent viscous damping can be used to evaluate the damping performance of nonlinear materials. The equivalent viscous damping [24,25], $C_{eq}$, is calculated by equating the dissipated energy over a cycle, $E_{d}$ to that of the viscous damping. The energy dissipated by a material is calculated using

(5) $$E_{d}=A_{h}t_{h}\oint \sigma d\epsilon =A_{h}t_{h}\int_{0}^{2\pi /\omega }\sigma \dot{\epsilon }dt$$

Here, *w* is the frequency. $A_{h}$ and $t_{h}$ are the cross-sectional area and thickness of the material sample, respectively. Then, the equivalent viscous damping is determined by

(6) $$\;C_{eq}=\frac{E_{d}}{\pi \omega \epsilon_{0}^{2}t_{h}^{2}}$$

Here, $\epsilon_{0}$ is the amplitude of the sinusoidal strain cycle.

**Figure 8:**
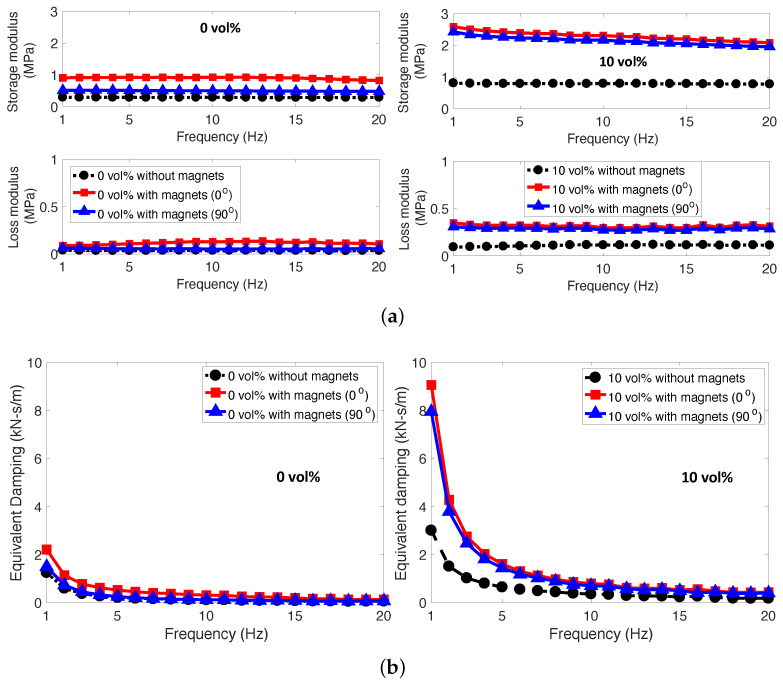
Complex modulus and equivalent viscous damping of the MREFs with Fe particle concentrations of 0 and 10 vol% in the frequency domain. (**a**) complex modulus; (**b**) equivalent viscous damping.

One example of the complex modulus and equivalent viscous damping of the MREFs in the frequency domain was presented in Figure 8. As shown in this figure, the storage and loss moduli of the MREFs were weakly dependent on the frequency, but, the equivalent viscous damping was strongly related to the frequency because the equivalent damping is inversely proportional to the frequency as seen in Equation (6). For a particle concentration of 0 vol%, the storage and loss moduli of all of the MREFs are almost unchanged with the frequency. At a particle concentration of 10 vol%, the loss modulus behavior of the MREFs is similar to the 0 vol% cases. However, the storage modulus for both the MREFs with magnets (0° and 90°) slightly decreased as the frequency increased. This decrease in the storage modulus may be due to the Mullins effect [26,27] or stress softening which is a common phenomenon of particle-filled elastomeric materials. So far, there is no unified explanation model for the Mullins effect, but Mullins [28] and Bouche [29,30] explained that this stress-softening is due to disentanglement of the polymer network chains produced by the breakdown of particle filler–matrix interactions. On the other hand, both the MREFs with magnets (0° and 90°) showed higher storage and loss moduli over the frequency range tested here than the MREFs without magnets. This implies that the MREFs with magnets (0° and 90°) have higher stiffness and damping capability than the MREFs without magnets. Also, the equivalent viscous damping of both the MREFs with magnets (0° and 90°) was higher than the MREFs without magnets. At a higher frequency range, the difference of the equivalent viscous damping between with magnets and without magnets became significantly smaller.

**Figure 9:**
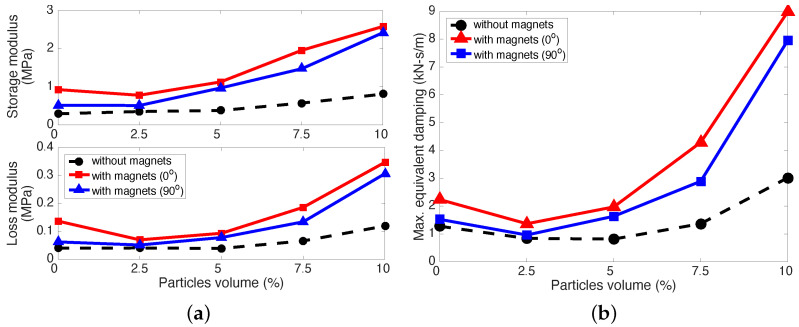
Maximum complex modulus and equivalent viscous damping of the MREFs versus particle concentration. (**a**) maximum complex modulus; (**b**) maximum equivalent viscous dammping.

Figure 9 presents the maximum complex modulus and maximum equivalent viscous damping of the MREFs in relation to particle concentration. As the particle concentration increased, there was a corresponding rise in both the maximum storage and loss moduli, along with their maximum equivalent damping. Up to a concentration of 5 vol%, the disparities in these performance metrics between the MREFs with magnets and the MREF without magnets were minimal. However, beyond 5 vol%, the gaps in these performance indices widened. Elevated Fe particle concentrations (>5 vol%) led to the MREFs adopting a more viscoelastic nature. In alignment with the quasi-static findings presented in Figure 5, Figure 6 and Figure 7, the MREF with the magnet (0°) exhibited superior dynamic compressive performance. Notably, the discrepancy in performance between the case with no-rotated magnet placement and the 90°-rotated magnet placement was less pronounced than observed in the quasi-static results.

## 4. Conclusions

The development of magnetorheological elastomeric foams (MREFs) as an adaptive cushioning material was discussed and their compressive properties were presented in this study. To this end, isotropic MREF which had no oriented particle structure was fabricated by randomly dispersing 1–10 micron-sized carbonyl Fe particles into an uncured silicone elastomeric foam in the absence of a magnetic field. The MREF samples were placed between two flexible multipole strip magnets which had arrays of north and south magnetic poles on one face. The quasi-static and dynamic compressive tests were conducted by using a servo-hydraulic test machine. To evaluate the effect of the particle concentrations, five different carbonyl Fe particle volume fractions (vol%) of 0, 2.5, 5, 7.5, and 10% were chosen. In addition, the variation of the compressive properties of the MREFs due to different magnet placement configurations (0° and 90°) was also experimentally investigated. The conclusions of this study are below.

- It was observed from the quasi-static experimental tests (the MREFs were compressed up to 65% strain) that both MREFs with magnets (0° and 90°) were as soft and flexible as the MREFs without magnets until low compressive strain (below 35% strain). But, at higher strain (after 35% strain), they became much firmer and stiffer.
- As the Fe particle concentration increased, the quasi-static compressive stresses and energy absorption densities of the MREFs continuously increased. However, the cushioning indices which were defined by the ratio of the stress at 65% strain to the stress at 25% strain showed a V-shaped incremental curve with the particle concentration.
- From dynamic experimental sweep tests (sweep frequency range was 1–20 Hz), it was found that the storage and loss moduli of the MREFs were very weakly frequency dependent. However, their equivalent dampings were strongly dependent on the frequency.
- After 5 vol% particle concentration, the maximum storage and loss moduli of the MREFs and their maximum equivalent dampings also greatly increased with the particle concentration.
- The no-rotated (0°) magnet placement case in which the top and bottom magnets had the same multipole direction produced higher quasi-static and dynamic compressive stresses than the 90°-rotated magnet placement case in which the multipole direction of the top and bottom magnets showed a 90° difference.

This study focused on examining isotropic MREF samples, where the carbonyl iron particles are uniformly distributed throughout the material. However, by applying a magnetic field during curing, the carbonyl iron particles can be aligned with the direction of the magnetic field, leading to the fabrication of anisotropic MREF samples. In the near future, the mechanical characteristics of anisotropic MREFs will be investigated.

## Data Availability

The data presented in this study are available on reasonable request from the corresponding author.

## References

[1] Deshmkh S., McKinley G. (2006). Adaptive Energy-Absorbing Materials Using Field-Responsive Fluid-Impregnated Cellular Solids. Smart Mater. Struct..

[2] D’Auria D., Davino D., Pantani R., Sorrentino L. (2016). Polymeric Foam-Ferromagnet Composites as Smart Lightweight Materials. Smart Mater. Struct..

[3] Yoo J.H., Wereley N.M. (2002). Design of a High-Efficiency Magnetorheological Valve. J. Intell. Mater. Syst. Struct..

[4] Pierce R., Choi Y.T., Wereley N.M. (2022). The Effect of Mesocarbon Microbeads on Magnetorheological Fluid Behavior. J. Intell. Mater. Syst. Struct..

[5] Mao M., Hu W., Choi Y.T., Wereley N.M., Browne A.L., Ulicny J. (2014). Experimental Validation of a Magnetorheological Energy Absorber Design Analysis. J. Intell. Mater. Syst. Struct..

[6] Choi Y.T., Robinson R., Hu W., Wereley N.M., Brichette T.S., Bolukbasi A.O., Woodhouse J. (2016). Analysis and Control of a Magnetorheological Landing Gear System for a Helicopter. J. Am. Helicopter Soc..

[7] Powers B.E., Choi Y.T., Wereley N.M. (2016). Analysis of Impact Loads in a Magnetorheological Energy Absorber Using a Bingham Plastic Model with Refined Minor Loss Factors Accounting for Turbulent Transition. Mechanica.

[8] Xie L., Choi Y.T., Liao C.R., Wereley N.M. (2016). Long Term Stability of Magnetorheological Fluids Using High Viscosity Linear Polysiloxane Carrier Fluids. Smart Mater. Struct..

[9] Choi Y.T., Xie L., Wereley N.M. (2016). Testing and Analysis of Magnetorheological Fluid Sedimentation in a Column Using a Vertical Axis Inductance Monitoring System. Smart Mater. Struct..

[10] Gong Q., Wu J., Gong X., Fan Y., Xia H. (2013). Smart Polyurethane Foam with Magnetic Field Controlled Modulus and Anisotropic Compression Property. RSC Adv..

[11] Carlson J.D., Jolly M.R. (2000). MR fluid, foam and Elastomer Devices. Mechatronics.

[12] Scarpa F., Bullough W.A., Lumley P. (2004). Trends in Acoustic Properties of Iron Particle Seeded Auextic Polyurethane Foam. IMechE Part C J. Mech. Eng. Sci..

[13] Wereley N.M., Perez C., Choi Y.T. (2018). Strain-Dependent Dynamic Compressive Properties of Magnetorheological Elastomeric Foams. AIP Adv..

[14] Muhazeli N.S., Nordin N.A., Ubaidllah U., Mazian S.A., Aziz S.A.A., Nazmi N., Yahya I. (2020). Magnetic and Tunable Sound Absorption Properties of an In-Situ Prepared Magnetorheological Foam. Materials.

[15] Choi Y.T., Wereley N.M. (2022). Adaptively Tunable Magnetorheological Elastomer-Based Vibration Absorber for a Propeller Aircraft Seat. AIP Adv..

[16] Park Y.J., Yoon J.Y., Kang B.H., Kim G.W., Choi S.B. (2020). A Tactile Device Generating Repulsive Forces of Various Human Tissues Fabricated from Magnetic-Responsive Fluid in Porous Polyurethane. Materials.

[17] (2024). What Are the Advantages of Silicone Foam over Polyurethane Foam?. https://mascherpa.it/en/blog/what-are-the-advantages-of-silicone-foam-over-polyurethane-foam/.

[18] Li W.H., Zhang X.Z., Du H. (2013). Magnetorheological Elastomers and Their Applications. Advances in Elastomers I. Advanced Structured Materials.

[19] Vatandoost H., Hemmatian M., Sedaghati R., Rakheja S. (2020). Dynamic Characterization of Isotropic and Anisotropic Magnetorheological Elastomers in the Oscillatory Squeeze Mode Superimposed on Large Static Pre-Strain. Compos. Part B.

[20] Robertson C.G., Wang X. (2005). Isoenergetic Jamming Transition in Particle-Filled System. Phys. Rev. Lett..

[21] Robertson C.G., Vaikuntam S.R., Heinrich G. (2020). A Nonequilibrium Model for Particle Networking/Jamming and Time-Dependent Dynamic Rheology of Filled Polymers. Polymers.

[22] Wu K., Zou J., Wang X. (2022). Impacts of Filler Loading and Particle Size on the Transition to Linear-Nonlinear Dichotomy in the Rheological Responses of Particle-Filled Polymer Solutions. J. Rheol..

[23] Tse F., Morse I., Hinkle R. (1978). Mechanical Vibrations: Theory and Applications.

[24] Brigley M., Choi Y.T., Wereley N., Choi S.B. (2007). Magnetorheological Isolators Using Multiple Fluid Modes. J. Intell. Mater. Syst. Struct..

[25] Brigley M., Choi Y.T., Wereley N. (2008). Experimental and Theoretical Development of Multiple Fluid Mode Magnetorheological Isolators. J. Guid. Control Dyn..

[26] Vakada K.C. (2005). Use of Advanced Material Modeling Techniques in Large-Scale Simulations for Highly Deformable Structures. Master’s Thesis.

[27] Gan R., Qi S., Zhao Y., Fu J., Li S., Li Y., Yu M. (2021). Magneto-Induced Mullins Effect of Anisotropic MRes under Compression Mode. Smart Mater. Struct..

[28] Mullins L. (1969). Softening of Rubber by Deformation. Rubber Chem. Technol..

[29] Bueche F. (1960). Molecular Basis of the Mullins Effect. J. Appl. Polym. Sci..

[30] Bueche F. (1961). Mullins Effect and Rubber-Filled Interaction. J. Appl. Polym. Sci..

